# EAM/FEMS launches *microLife*

**DOI:** 10.1093/femsml/uqaa001

**Published:** 2020-07-08

**Authors:** Axel Brakhage, Carmen Buchrieser, Matthias Horn, Paula Traktman

**Affiliations:** Microbiology and Molecular Biology, University of Jena, Germany; Institut Pasteur, Paris, France; Microbial Symbioses and head of the Department of Microbiology and Ecosystem Science, University of Vienna, Austria; Dean of the College of Graduate Studies and the Hirschmann Endowed Professor, Medical University of South Carolina, Charleston, SC, USA

We are excited to announce the launch of ***microLife***, the new open access journal of the European Academy of Microbiology (EAM, http://europeanacademyofmicrobiology.org/).

Microbiology has become one of the most influential sciences affecting health, the environment, the climate and also the economy. The latter is particularly evident in the recent corona virus epidemic as well as the emergence of multi-resistant microorganisms, which not only affect our daily lives but also have severe consequences for the global economy. The recent recognition of microbiomes as key determinants of plant, animal, human and ecosystem health illustrates the importance of our discipline and calls for intensified collaborative and interdisciplinary efforts to address global challenges of the 21st century.

We therefore anticipate that the number of top-tier papers based on microbial research with impact on a wide range of research areas will further increase. To accommodate the projected research output in this area, the EAM, supported by the Federation of European Microbiological Societies (FEMS), has decided to instate a new journal that provides a home for such top class research. Currently, FEMS provides a variety of microbiology journals, but a broad-scope journal with a remit to publish cross-disciplinary research of the highest quality is urgently needed.

***microLife*** is a **gold open access journal in microbiology**. Thus all content will be available for free immediately on the journal's website (https://academic.oup.com/microlife). We invite outstanding papers of the highest standard, novelty, and significance in all areas of microbiology as full-length research articles, short reviews or commentaries. ***microLife*** will cover all aspects of microbiology ranging from molecular and evolutionary biology, physiology and genetics, environmental microbiology, chemical biology of microorganisms, microbiome research, infection and host-responses to translational microbiology and microbial biotechnology. All microbes, bacteria, archaea, viruses, parasites, protists or fungi will be covered by ***microLife***.

***microLife*** welcomes direct submissions and also offers the Academy Submission route for EAM contributions. Submitting to ***microLife*** offers several benefits: The journal will allow format-neutral initial submissions and posting on preprint servers. Manuscripts considered for publication will not be rejected if similar results are published elsewhere by others after submission to ***microLife***. We will also consider manuscripts that confirm or extend a recently published study reporting major advances in the field. We will furthermore offer the option to recognize the work done by co-reviewers during peer review. The EAM member track includes the option to submit one manuscript per year, with EAM members acting as self-editing corresponding authors (see guidelines at https://academic.oup.com/microlife/pages/General_Instructions).

***microLife* was designed by members of our scientific community to serve the needs of our community**. As the show case journal of the EAM, ***microLife*** aims at establishing itself as one of the top journals in microbiology and its related fields, helping to further promote our scientific discipline. EAM members serve as editors of the journal further ensuring highest quality of the research published in ***microLife***. FEMS and our publisher, Oxford University Press, contribute their long-standing expertise in scientific publishing to this endeavour and will guarantee efficient promotion and communication of the research published in ***microLife***. Importantly, ***microLife*** as a journal of the Federation of European Microbiological Societies will direct profits to support young researchers for attending conferences or carrying out research visits in other laboratories. We are very proud of this agreement.

We invite you to join us on this exciting mission to establish ***microLife*** as a top class journal and look forward receiving your best research as submitted manuscripts.

## Notes

Axel Brakhage, Carmen Buchrieser, Matthias Horn, Paula Traktman

Editors-in-Chief (https://academic.oup.com/microlife)

*Axel Brakhage is Professor of Microbiology and Molecular Biology at the University of Jena, Germany, and the Director of the Leibniz Institute for Natural Product Research and Infection Biology (Leibniz-HKI) in Jena, Germany. His research focuses on the infection biology of human pathogenic fungi and the molecular biotechnology and ecology of natural products predominantly from fungi*.

*Carmen Buchrieser is Professor at the Institut Pasteur, Paris, France. Her research focuses on bacterial infections with particular interest in intracellular human pathogens. She studies genetic factors conferring bacterial virulence, their functions in host pathogen interactions, their regulation, their acquisition and their evolution*.

*Matthias Horn is Professor of Microbial Symbioses and head of the Department of Microbiology and Ecosystem Science at the University of Vienna, Austria. His research focuses on the biology and evolution of intracellular microbes. He studies the ecology of host-associated microbes, mechanisms of microbe–host interactions and microbial genome evolution*.

*Paula Traktman is the Dean of the College of Graduate Studies and the Hirschmann Endowed Professor at the Medical University of South Carolina in Charleston, SC, USA. Her virology research is focused on poxviruses, with a particular interest in genome replication and repair, the biogenesis of the viral membrane and the cell biology of the viral life cycle*.

